# Metacarpal Pain Unveiled: A Case Report and Literature Review of Dietrich’s Disease in Adolescence

**DOI:** 10.7759/cureus.57214

**Published:** 2024-03-29

**Authors:** Akhileshwar R Ginnaram, Shruti Kumar, Heta B Ladumor, Shilpa Mohanan, Janice W Murphy

**Affiliations:** 1 Diagnostic Radiology, University of Arkansas for Medical Sciences, Little Rock, USA; 2 Pediatric Radiology, University of Arkansas for Medical Sciences, Little Rock, USA

**Keywords:** musculoskeletal mri, pediatric radiology, pediatric orthopedics, adolescent hand injuries, adolescents, dietrich's disease, metacarpal pain, avascular necrosis (avn)

## Abstract

Dietrich's disease, also known as Mauclaire’s disease, is a rare condition characterized by avascular necrosis of the metacarpal heads, predominantly affecting adolescents. This case report aims to elucidate the diagnostic process and management of Dietrich's disease in adolescents.

A 15-year-old male adolescent presented with left ring finger metacarpophalangeal joint pain and restricted range of motion following a remote history of sports-related trauma. Clinical examination revealed tenderness and limited flexion at the affected joint. Radiographic evaluation demonstrated characteristic features of Dietrich's disease, including lucency and loss of height in the fourth metacarpal head and volar subluxation of the ring finger.

Computed tomography and magnetic resonance imaging (MRI) confirmed the diagnosis, revealing flattening of the metacarpal head, subchondral marrow edema, and joint effusion consistent with avascular necrosis.

The pathogenesis of Dietrich's disease remains incompletely understood, likely involving acquired deficits in arteriolar blood supply. Radiographic and MRI findings aid in diagnosis, distinguishing it from other conditions such as chondroblastoma and osteomyelitis. Treatment options range from conservative management to surgical interventions, depending on the severity of symptoms.

Dietrich's disease, though rare, should be considered in adolescents presenting with metacarpal pain and predisposing factors such as trauma or steroid use. Recognition of characteristic imaging features is essential for accurate diagnosis and appropriate management in adolescent populations. This case highlights the importance of early detection and multidisciplinary management in adolescents with Dietrich's disease to optimize outcomes and preserve hand function.

## Introduction

Dietrich's disease [1], also known as Mauclaire’s disease [2], is a rare medical condition characterized by avascular necrosis of the metacarpal heads. This condition primarily affects individuals between the ages of 13 and 18, with a higher prevalence in males [3-6]. Predisposing factors include a history of trauma, chronic steroid use, and systemic lupus erythematosus (SLE), though it is often considered idiopathic [7]. The disease’s predominant mechanism of action is linked to defects in healing, resulting in avascular malformation, acquired ischemia, and subsequent necrosis [3,5,7,8].

Given its rarity, Dietrich's disease poses a considerable challenge. However, early detection is crucial for optimal management [8,9]. In this report, we present a case study outlining the diagnosis, clinical presentation, imaging findings, and treatment options for a 15-year-old male patient diagnosed with Dietrich's disease.

## Case presentation

A 15-year-old male patient presented with complaints of pain and restricted range of motion in his left ring finger metacarpophalangeal (MCP) joint. The patient’s history includes a documented trauma incident one year earlier where his left hand was stepped upon during a football game. Complicating the clinical picture, the patient is asthmatic and being treated with inhaled corticosteroids. The patient denied experiencing numbness or tingling in the affected digit or extremity. Noteworthy risk factors in this case included a prior history of trauma and the ongoing use of chronic steroids.

Clinical examination revealed tenderness to palpation and limited flexion at the fourth MCP joint of the left ring finger. Importantly, the range of motion in the other digits of the affected hand was normal, and there were no signs of neurovascular compromise. A routine blood investigation showed no significant abnormalities.

**Figure 1:**
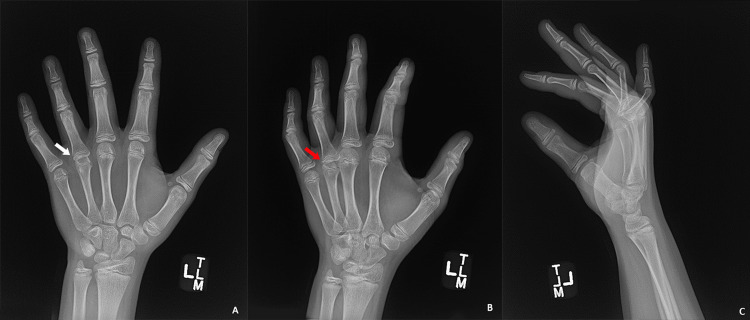
Plain film radiographs of the patient's left hand (a) Anterior-posterior view. Lucency and loss of height of the fourth metacarpal head (white arrow). (b) Oblique and (c) lateral views. Volar subluxation of the fourth MCP joint (red arrow) MCP: metacarpophalangeal

A radiographic evaluation of the left hand showed lucency and a loss of height in the head of the fourth metacarpal. This was initially attributed to a presumed prior fracture resulting from the documented antecedent trauma. Additionally, radiographs showed volar subluxation of the ring finger at the fourth MCP joint, a noteworthy feature illustrated in Figure 1.

**Figure 2:**
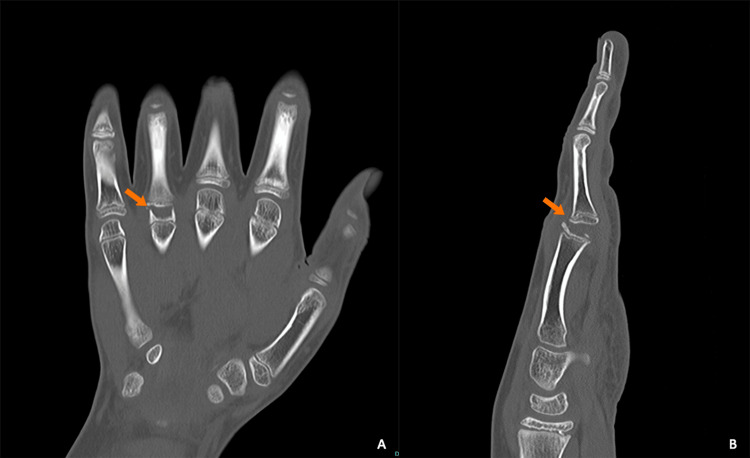
CT images of the left hand focusing on the fourth MCP joint in the bone kernel (a) Coronal CT of the left hand (bone kernel) displaying flattening and irregularity of the left metacarpal head (orange arrow). (b) Sagittal CT (bone kernel) depicting associated volar subluxation of the proximal phalanx (orange arrow) CT: computed tomography, MCP: metacarpophalangeal

**Figure 3:**
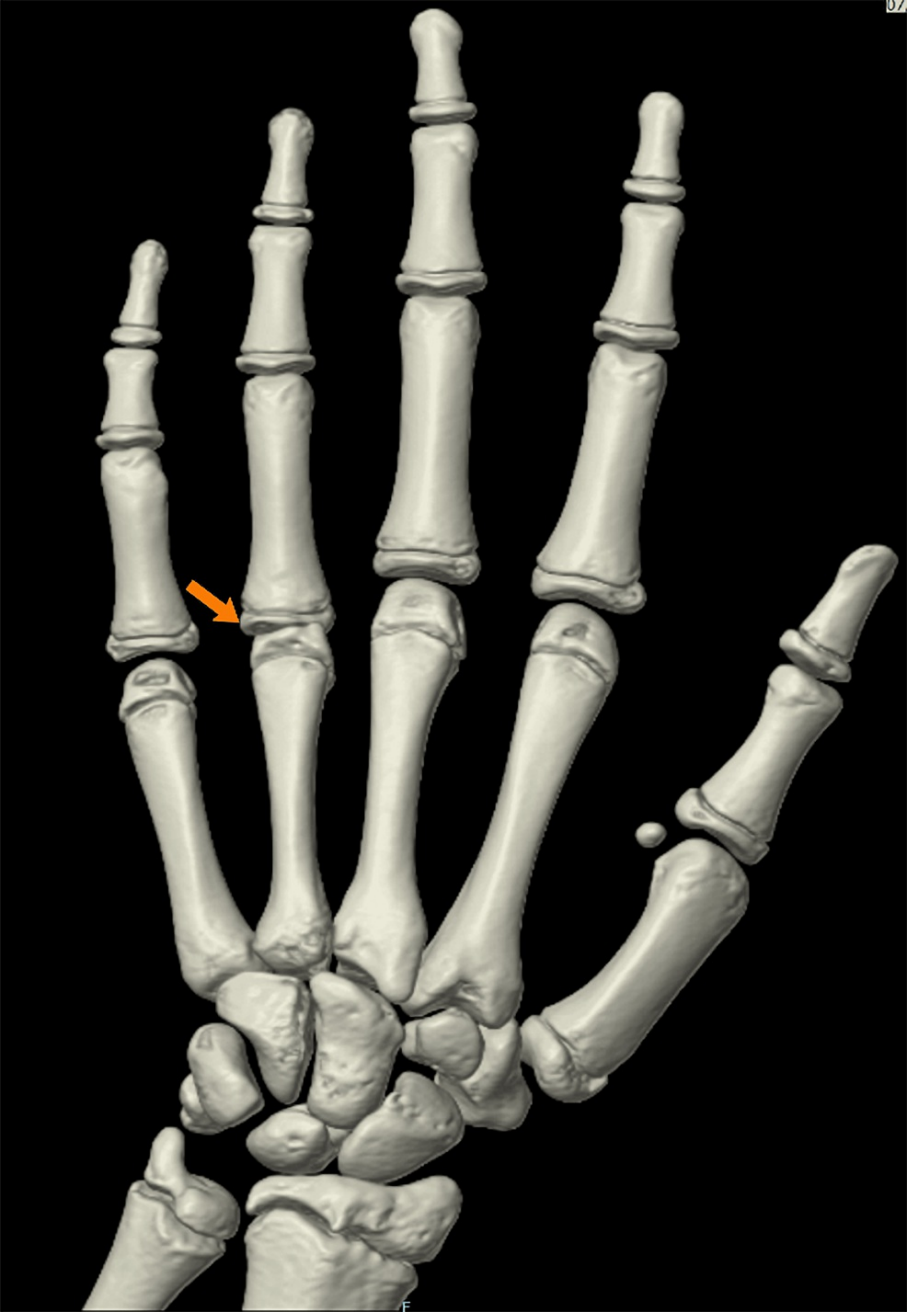
3D reconstruction of the left hand and wrist Shows irregularity with flattening of the fourth metacarpal epiphysis (orange arrow) 3D: three dimensional

To gain a more detailed understanding of the deformity and assess the potential presence of a suspected pathologic fracture, a computed tomography (CT) scan of the hand and wrist was performed with 3D reconstructions (Figures 2-3), which confirmed the deformity within the epiphysis of the fourth metacarpal and volar subluxation of the proximal phalanx of the fourth digit.

**Figure 4:**
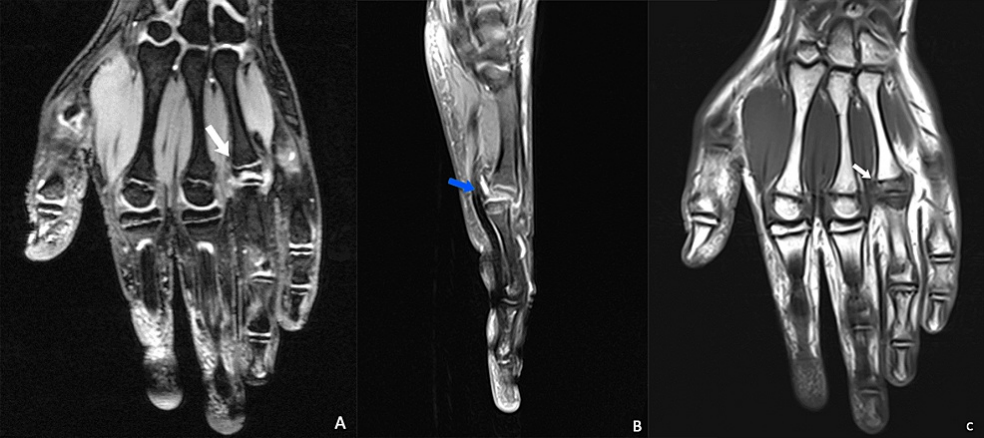
MRI imaging of the left hand and wrist (a) Coronal PD-FS and (b) sagittal T2 weighted images of the left hand depict subchondral marrow edema in the fourth metacarpal head (white arrow), with minimal joint fluid and volar displacement of the proximal fourth phalanx over the abnormal metacarpal head (blue arrow). (c) Coronal T1 weighted image of the left hand shows flattening and irregularity of the fourth metacarpal head with hypointense marrow signal in the subchondral bone (white arrow) MRI: magnetic resonance imaging

Subsequent MRI studies revealed flattening of the fourth metacarpal head with irregularities in the underlying cortex. Additional findings included subchondral marrow edema, effusion within the fourth MCP joint, and confirmed subluxation of the proximal phalanx of the fourth digit (Figure 4). These MRI findings strongly suggested avascular necrosis of the fourth metacarpal head or Dietrich's disease.

Despite the diagnostic challenges posed by the condition, the patient maintained excellent functional status, prompting the healthcare team to adopt a conservative treatment approach rather than more invasive surgical interventions.

## Discussion

Dietrich’s disease is an uncommon pathology characterized by avascular necrosis of the metacarpal head, initially described by Mauclaire and/or Dietrich in the 1930s [2,10]. This condition commonly affects the middle finger (46% of cases), followed by equal involvement of the index and ring fingers (19%), the little finger (12%), and least commonly, the thumb (5%) [7,11].

The underlying pathogenesis of the disease remains incompletely understood. A proposed etiology implicates a deficit in the pericapsular and central arteriolar blood supply to the metacarpal, especially concerning the preponderance of single-vessel intraosseous blood supply to metacarpal distal epiphyses [3,8,9]. McElfresh and Dobyns (1983) describe a series of 100 patients with multiple intraarticular metacarpal head fractures and associated abnormalities, where the etiology of Dietrich’s disease follows a similar pattern to other more obvious intraarticular fractures [4]. Weissman et al. report multiple radiographic findings in SLE, particularly in lupus vasculitis, where end arteriolar occlusion leading to avascular necrosis is depicted [12]. It shows similar findings to those that are seen in our case on MRI, as well as other similar cases with associated MRI imaging [3,5,6]. The complications include early osteoarthritic involvement of the MCP joints.

Radiographic findings in Dietrich’s disease typically show a deformity of the metacarpal head with mixed lytic and sclerotic areas within the subchondral bone, which are better characterized on CT. Also described in the literature is the increased density of the entire epiphysis, followed by degenerative (possibly early osteoarthritic) changes in the associated region [6,10].

MRI plays a role in the early detection of the disease and demonstrates flattening of the metacarpal head with cortical irregularity, subchondral marrow edema, and joint effusion. On MRI, avascular necrosis is typically characterized by T1-weighted images that exhibit reduced signal intensity against the backdrop of hyperintense fatty marrow. Diffusion-weighted sequences show increased diffusion [13,14]. Additionally, T2-weighted sequences reveal a unique pattern of a second hyperintense inner line between normal marrow and ischemic marrow known as the double-line sign [5,15]. It may also be used to determine the area of an associated necrotic bone. A nuclear medicine bone scan may be useful to identify early/developing avascular necrosis before anatomic findings are apparent [15,16].

Differential diagnoses, particularly with pediatric epiphyseal injuries, include chondroblastoma and osteomyelitis. Chondroblastoma presents as a well-defined expansile lytic lesion with thin sclerotic margins and internal “fluffy” calcifications on radiographs and CT. On MRI, a thin hypointense sclerotic rim is seen on T1- and T2-weighted imaging with surrounding bone edema [15].

Osteomyelitis is characterized by focal osseous erosions or cortical loss, periosteal reaction, soft tissue swelling, and blurring of fat planes on radiographs. Associated T1 hypointensity, postcontrast enhancement, diffusion restriction, and a high signal on fluid-sensitive sequences are the hallmarks of osteomyelitis on MRI [14,15]. Particularly, diffusion sequences are quite helpful for differentiating avascular necrosis from osteomyelitis, owing to their opposing signal restriction characteristics [13,14].

Treatment options vary, from conservative medical management to surgical options. Conventional treatment includes rest and NSAIDs to control pain and inflammation. Upon failure of medical management, surgical options may be explored [6,15]. Some of the surgical options include debridement of avascular necrotic bone with subsequent cancellous bone grafts, osteochondral mosaicplasty reconstruction, surgical decompression, autograph transplantation, and flexion osteotomy of the head of the metacarpal with metacarpal head reversal [3,6,15,17]. These approaches may be considered viable options in the event of medical management failure.

## Conclusions

Dietrich's disease, although rare, should be included as a radiological consideration in patients, particularly adolescents, presenting with metacarpal pain and a history of trauma and/or steroid use. Diagnostic confidence can be strengthened by recognizing imaging features such as metacarpal bone flattening and marrow signal changes on MRI.

## References

[1] Dietrich H (1932). The subchondral focal disease of the metacarpal (Article in German). Langenbecks Arch Klin Chir.

[2] Green DP (2011). Mauclaire's disease. J Hand Surg Am.

[3] Sagar P, Shailam R, Nimkin K (2010). Avascular necrosis of the metacarpal head: a report of two cases and review of literature. Pediatr Radiol.

[4] McElfresh EC, Dobyns JH (1983). Intra-articular metacarpal head fractures. J Hand Surg.

[5] Aldekhayel S, Ghanad E, Mudgal CS (2018). Avascular necrosis of the metacarpal head: a review of 4 cases. J Hand Surg Am.

[6] Wijeratna MD, Hopkinson-Woolley JA (2012). Conservative management of Dieterich disease: case report. J Hand Surg Am.

[7] McGoldrick NP, McGoldrick FJ (2015). Avascular necrosis of the metacarpal head: a case of Dietrich's disease and review of the literature. Am J Case Rep.

[8] Wright TC, Dell PC (1991). Avascular necrosis and vascular anatomy of the metacarpals. J Hand Surg Am.

[9] De Smet L (1998). Avascular necrosis of the metacarpal head. J Hand Surg.

[10] Hagino H, Yamamoto K, Teshima R, Kishimoto H (1990). Sequential radiographic changes of metacarpal osteonecrosis. A case report. Acta Orthop Scand.

[11] Saliba T, Simoni P, Boitsios G (2023). A painful finger-answer: Dieterich's disease. Skeletal Radiol.

[12] Weissman BN, Rappoport AS, Sosman JL, Schur PH (1978). Radiographic findings in the hands in patients with systemic lupus erythematosus. Radiology.

[13] Ahmed N, Sriskandarajah P, Burd C, Riddell A, Boyd K, Kaiser M, Messiou C (2019). Detection of avascular necrosis on routine diffusion-weighted whole body MRI in patients with multiple myeloma. Br J Radiol.

[14] Lee YJ, Sadigh S, Mankad K, Kapse N, Rajeswaran G (2016). The imaging of osteomyelitis. Quant Imaging Med Surg.

[15] Fan XL, Wang WT, Wang J, Xiao R (2023). Current management of avascular necrosis of the metacarpal head: a comprehensive literature review. Int J Surg.

[16] Maryada VR, Joseph VM, Pancholi S, Reddy AV (2018). Dieterich disease treated with curettage and bone grafting: a case report. J Orthop Case Rep.

[17] Maes M, Hansen L, Cheyns P (2010). Osteochondral mosaicplasty as a treatment method for bilateral avascular necrosis of the long finger metacarpal: case report. J Hand Surg Am.

